# An *aroD* Ochre Mutation Results in a *Staphylococcus aureus* Small Colony Variant That Can Undergo Phenotypic Switching via Two Alternative Mechanisms

**DOI:** 10.3389/fmicb.2017.01001

**Published:** 2017-05-31

**Authors:** Ping Zhang, John A. Wright, Ahmed A. Osman, Sean P. Nair

**Affiliations:** ^1^Department of Microbial Diseases, UCL Eastman Dental Institute, University College LondonLondon, United Kingdom; ^2^Immunology Catalyst, GlaxoSmithKline plcStevenage, United Kingdom

**Keywords:** *Staphylococcus aureus*, small colony variants, ochre mutation, phenotypic switching, *aroD*

## Abstract

*Staphylococcus aureus* can undergo phenotypic switching between a normal colony phenotype (NCP) and a small colony variant (SCV). The SCV phenotype confers increased antibiotic resistance and the capacity to persist within human tissues and cells, and because these cells can revert back to the NCP they cause chronic and/or recurrent infections that are very difficult to treat. A complete picture of the genetic events that can lead to phenotypic switching in *S. aureus* is currently lacking. We describe the selection of an SCV with a previously unreported genetic alteration leading to an ochre mutation of *aroD*. In addition to the known mechanisms of phenotypic switching between the SCV and the NCP we describe a previously unreported mechanism involving tRNA ochre suppressors arising. The ochre suppressor strains had wild-type growth rates and restored antibiotic sensitivity, similar to the wild-type strain. However, whilst they had increased virulence compared to the SCV parent strain, their virulence was not restored to that of the NCP parental strain. These findings establish that phenotypic switching between the NCP and SCV states can give rise to strains with different pathogenic potential.

## Introduction

*Staphylococcus aureus* small colony variants (SCVs) are a naturally arising slow-growing subpopulation of *S. aureus*. They are termed SCVs because their colonies are about 1/10 the size of normal colonies on agar after 24 h of growth. *S. aureus* SCVs are frequently associated with persistent and recurrent infections, such as osteomyelitis, implant infections and airway infections in cystic fibrosis patients (von Eiff et al., 2006), or patients receiving long-term antibiotic treatment (Proctor et al., 1995; von Eiff et al., 1997a, 2001). This is in part because SCVs can persist within non-phagocytic host cells such as epithelial cells (Atalla et al., 2010), endothelial cells (von Eiff et al., 1997b) and bone cells (Wright and Nair, 2010). This is because SCVs have reduced toxin production and do not induce an immune response (Tan et al., 2014; Ou et al., 2016). In addition genes responsible for adhesion and biofilm formation are generally upregulated in SCVs (Proctor et al., 2006), enhancing the capacity of the bacterium to persist in implant infections, cystic fibrosis patients, and osteomyelitis.

*Staphylococcus aureus* SCVs isolated from patients are often found to be auxotrophic for metabolites such as heme, menadione, or thymidine (Proctor et al., 2006). The auxotrophic nature of SCVs, generally, seems to relate to the antibiotic treatment that the patient was receiving with aminoglycosides giving rise to heme and menadione auxotrophs (Balwit et al., 1994), while trimethoprim-sulfamethoxazole treatment gives rise to thymidine auxotrophs (Kriegeskorte et al., 2015). A number of studies have shown that SCVs can also be generated by exposure to very low levels of triclosan (Bayston et al., 2007; Seaman et al., 2007; Latimer et al., 2012). However, the auxotrophy of these SCVs was not defined and the likelihood of them arising *in situ* in clinical settings is questionable because of the levels of triclosan in hand washes and other cleaning products (Seaman et al., 2007; Latimer et al., 2012). Heme and menadione are required for the synthesis of cytochromes and menaquinone, respectively. These molecules are essential for respiration and thus SCVs defective in the biosynthesis of either of these two molecules switch to generating energy via fermentation resulting in reduced growth and bacterial numbers, they have a decreased bacterial membrane potential, and thus a decreased electrochemical gradient, which is associated with increased resistance to antimicrobials (Sendi and Proctor, 2009; Mike et al., 2013). Thymidine auxotrophs have similar phenotypes to heme and menadione auxotrophs due to decreased TCA cycle activity, which results in down-regulation of the ETC (Proctor et al., 2014).

The SCV phenotype is usually unstable and phenotypic switching back to the normal colony phenotype (NCP) occurs (Massey et al., 2001; Kahl, 2014). This phenotypic switching may explain why SCV infections recur after periods of apparent remission (Kahl, 2014). Whilst numerous studies on *S. aureus* SCVs have been published, there are only a few studies that show the genetic basis for phenotypic switching back to the NCP and the frequency of reversion (Vestergaard et al., 2016). It was reported that phenotypic switching from SCV to NCP restored antibiotic sensitivity and haemolytic activity to that of the wild-type (Lannergård et al., 2008; Dean et al., 2014). Given the nature of the reversions one might predict that other phenotypes, such as the capacity to form biofilms and virulence, would be restored to normal wild-type levels, though this was not examined.

In this study, we isolated a spontaneous menadione auxotrophic SCV to determine the genetic basis of SCV reversion to NCP and the consequences of this phenotypic switching on antibiotic resistance, biofilm formation, and virulence. We determined that this SCV had a point mutation in the *aroD* gene, which has not been previously reported. The gene encodes 3-dehydroquinate dehydratase, an enzyme participating in the shikimate pathway that catalyzes the conversion of 3-dehydroquinate to 3-dehydroshikimate, the third step in the shikimate pathway leading to the synthesis of chorismate. Chorismate is a precursor for the synthesis of a range of aromatic compounds, including aromatic amino acids and menaquinone. As expected, this SCV is auxotrophic for all aromatic amino acids and menaquinone. Two classes of revertant arose upon phenotypic switching to the NCP, those in which the *aroD* gene mutation was reversed and those that had a compensatory mutation in one of two tyr-tRNA genes.

## Materials and Methods

### Strains, Plasmids, and Oligonucleotides

The bacterial strains and plasmids used in this study are summarized in **Tables 1, 2**, respectively. The oligonucleotides used are listed in Supplementary Table S1. *E. coli* was grown under aerobic conditions in Luria-Bertani (LB) broth or LB agar (supplemented with 10 μg/ml ampicillin to maintain plasmids when required) at 37°C. *S. aureus* was grown under aerobic conditions in tryptic soy broth (TSB) or tryptic soy agar (TSA) at 37°C. Appropriate antibiotics (erythromycin at 5 μg/ml or chloramphenicol at 10 μg/ml) were supplemented if the strains carried antibiotic markers. The *S. aureus* SCV, SCV445, was grown in the presence of 50 μg/ml kanamycin to select against phenotypic switching to the NCP.

**Table 1:** Bacterial strains used in this study.

Strains	Properties	Reference
*E. coli* DH5α	Cloning host	Invitrogen
*S. aureus* RN4220	Restriction deficient strain derived from *S. aureus* 8325-4	Kreiswirth et al., 1983
*S. aureus* LS-1	*S. aureus* septic arthritis isolate from mice	Bremell et al., 1990
*S. aureus* 8325-4	Wild-type laboratory strain	Novick, 1967
*S. aureus* 8325-4 *menD*	*S. aureus* 8325-4 with *ermC* gene inserted in *menD*	Bates et al., 2003
*S. aureus* SCV445	Spontaneous SCV derived from *S. aureus* LS-1 with mutation in *aroD*	This study
*S. aureus* LS-1 *aroD*	*S. aureus* LS-1 with nucleotides 448–717 bp deleted in *aroD*	This study
*S. aureus* LS-1 *menD*	*S. aureus* LS-1 with *erm* gene inserted in *menD*	This study
*S. aureus* LS-1 Δ*hemB*	*S. aureus* LS-1 derivative Δ*hemB*	Wright and Nair, 2012
PZ160-163	Revertant of SCV445 with a wild-type *aroD* gene	This study
PZ164	A revertant of SCV445 carrying mutation in tyr-tRNA.	This study
PZ165	A revertant of SCV445 carrying mutation in tyr-tRNA	This study

**Table 2:** Plasmids used in this study.

Plasmid	Characteristics	Reference
pKOR1	Inducible promoter *Pxyl/tetO* controlling antisense *secY*, lambda recombination sites, *attp*, flanking a *ccdB* gene. Amp^r^ (G- selection), Cm^r^ (G+ selection)	Bae and Schneewind, 2006
pSK236	Shuttle vector containing pUC19 cloned into the HindIII site of pC194. Amp^r^ (G- selection), Cm^r^ (G+ selection)	Gaskill and Khan, 1988
pHCMC05	Shuttle vector containing inducible promoter *lacI-Pspac*	Nguyen et al., 2005
pPZ138-7	pKOR1 derivative with *ccdB* replaced by the upstream and downstream sequences of *S. aureus aroD* gene	This study
pPZ137-3	pSK236 derivative with *Pspac-aroD* cloned in multiple cloning site	This study
pAAO1	pUC57 derivative containing a synthetic *aro*D gene (*aro*D_tyr_) with a 450A > T change compared to the wild-type. The gene was flanked by *Bam*HI and *Xba*I.	Genscript (this study)
pSK236::*aroD*_tyr_	pSK236 derivative with *Pspac*-*aro*D*tyr* cloned in multiple cloning site	This study

*G+, Gram-positive; G-, Gram-negative; Amp^*r*^, ampicillin-resistant; Cm^*r*^, chloramphenicol-resistant.*

### Growth Experiments in TSB

For the characterization of *S. aureus* growth dynamics in TSB, a starter culture was prepared by inoculating a single colony of *S. aureus* into 5 ml TSB containing appropriate antibiotics and grown for 16 h. The optical density at 600 nm (OD_600__nm_) of the starter culture was measured using a Pharmacia Biotech Ultrospec 2000 UV/Visible Spectrophotometer (GE Healthcare Life Sciences, Buckinghamshire, United Kingdom), then diluted in TSB supplemented with appropriate antibiotics and/or indicated substance to a calculated OD_600__nm_ of 0.05. This was then incubated at 37°C with shaking at 200 rpm. Growth was monitored by measuring the OD_600__nm_ at 1-h intervals, or by determining colony-forming units (CFU/ml) every 3 h by spreading serial dilutions of the culture onto TSA and incubating aerobically at 37°C for 24–48 h before colonies were counted.

### Growth Experiments in Chemically Defined Media (CDM)

The CDM used to characterize *S. aureus* growth dynamics was adapted from a described recipe (Pattee and Neveln, 1975), the constituents are listed in Supplementary Table S2. A starter culture was prepared by inoculating a single colony of *S. aureus* into 5 ml TSB containing appropriate antibiotics, which was then grown for 16 h. The cells were collected by centrifugation at 2500 ×*g* for 5 min, washed once with phosphate buffered saline (PBS, pH = 7.4), and re-suspended in 5 ml of the CDM used for growth experiments. The OD_600__nm_ of the suspension was measured, followed by diluting into CDM to a calculated OD_600__nm_ of 0.05. The remaining steps were as described for the growth experiments in TSB.

### DNA Sequencing

Genomic DNA from *S. aureus* strains was prepared from overnight cultures using QIAamp DNA Mini Kit (Qiagen). Overnight cultures of SCV445 were grown in TSB with 50 μg/ml of kanamycin, an aliquot of which was plated out on TSA to establish that none of the cell population had undergone phenotypic switching from SCV to NCP, before the remaining culture was used to obtain DNA. All DNA fragments for sequencing were amplified using Vent DNA polymerase (New England Biolabs). The primers used for PCR amplification and sequencing are listed in Supplementary Table S3. Sanger sequencing was performed by Genewiz UK Limited.

### Construction of *aroD* and *menD* Mutants

Deletion of the *aroD* gene in *S. aureus* LS-1 was performed according to the method of Bae and Schneewind (2006) and as previously described (Wright and Nair, 2012). A deletion from nucleotide 448 bp to 717 bp was constructed by amplifying regions flanking the gene using primer pairs P194/P195 and P196/P197.

A *S. aureus* LS-1 *menD* mutant was constructed by homologous recombination. A 3.1 kb fragment containing *menD* disrupted by an *ermC* insertion was amplified from *S. aureus* 8325-4 *menD*::*ermC* (Bates et al., 2003) using primers P125 and P126. The amplified fragment was treated with T4 polynucleotide kinase and ligated to pKOR1 which had been digested with *Eco*RI and *Eco*RV and treated with Klenow fragment. Subsequent steps were as described previously (Wright and Nair, 2012). The insertion of *ermC* into *menD* in the chromosome of LS-1 was confirmed by PCR amplification using primers P125 and P128.

### Complementation of the *aroD* Mutant

To complement the *aroD* mutant, the *aroD* gene from *S. aureus* LS-1 was amplified using primers P192 and P193. The PCR product was digested using *Bam*HI and *Xba*I. The *lacI-Pspac* region of plasmid pHCMC05 was excised (Nguyen et al., 2005) using *Sac*I and *Bam*HI, and together with the *aroD* fragment was ligated with *Sac*I-*Xba*I digested pSK236 to give plasmid pPZ137-3, which was introduced into the *aroD* mutant as previously described (Wright and Nair, 2012). In addition a synthetic *aroD* gene, with a base change at 448 of A to T, flanked by *Bam*HI and *Xba*I sites was synthesized by Genscript and cloned as described above to complement the *aroD* mutant with a mutated *aro*D gene (*aroD*tyr) coding for a K149Y substitution.

### Characterization of SCV445 Auxotrophism

Disk diffusion assays were used to determine the auxotrophic phenotype of SCV445 as previously described (Bauer et al., 1966). Heme (Fluka), menadione (Sigma), and thymidine (Fluka) were prepared at 1, 10, 100, and 1000 μg/ml, along with negative controls, and 15 μL of each substance was inoculated on to a disk and placed in the center of a TSA plate onto which SCV445 had been spread.

### Susceptibility to Antimicrobial Agents

The minimum inhibitory concentration (MIC) of kanamycin against *S. aureus* LS-1, SCV445 and the phenotypically switched strains PZ164 and PZ165 were determined in TSB according to a protocol described previously (Wiegand et al., 2008) and following CLSI guidelines (M07-A10). Briefly, an overnight culture was prepared by growing a single colony of *S. aureus* in 5 ml TSB for 16 h. The culture was diluted in fresh TSB to approximately 1 × 10^6^ CFU/ml. Aliquots of 50 μl were added to an equal volume of TSB containing antibiotics at a range of concentrations 0, 1, 2, 4, 8, 16, 32, 64, 128, 256, 512, and 1024 μg/ml in polystyrene 96-well plates (Thermo Scientific NUNC). The microtiter plate was incubated at 37°C with shaking at 200 rpm. The MIC was defined as the lowest concentration of antibiotic that resulted in no detectable bacterial growth.

### Measurement of the Frequency of Phenotypic Switching

The frequency of SCV445 phenotypic switching to the NCP was measured in fluctuation tests as originally described by Luria and Delbrück (1943). A colony of SCV445 on TSA containing 50 μg/ml kanamycin was inoculated into 10 ml TSB containing 50 μg/ml kanamycin, followed by incubation at 37°C with agitation at 200 rpm overnight. The 100 μl aliquots 10-fold serial dilutions of the culture were spread on TSA and incubated at 37°C. TSA plates were visually scanned for fast-growing NCP colonies after 24 and 48 h of incubation. The mutation rate was calculated according to the formula μ = -(1/*N*) ln *P*_0_, where μ is the mutation rate per cell per generation, *N* is the total number of colonies counted, and *P*_0_ is the proportion of SCV phenotype colonies. Visually identified NCP colonies were streaked on TSA and compared to the wild-type LS-1 to confirm the normal-growth phenotype.

### Biofilm Formation

*Staphylococcus aureus* was grown overnight in TSB, pelleted by centrifugation and resuspended to an OD_600__nm_ of 0.02 in BHI containing 1% glucose. Bacterial suspensions were inoculated into 96 well microtiter plates (200 μl per well) and incubated statically for 24 h at 37^o^C. Plates were washed three times with PBS and stained with 0.1% crystal violet for 5 min. The plates were rinsed with water to remove excess dye and then dried. The dye was solubilized by adding 200 μl of 33% glacial acetic acid to each well and incubated for 10 min with shaking. Biomass was measured by reading the absorbance at 590 nm. For determination of *P*-values, paired Student’s *t*-tests were performed.

### *Galleria mellonella* Virulence Assay

The *Galleria mellonella* virulence assay was performed essentially as described previously (Peleg et al., 2009) with slight modification. *Galleria mellonella* (obtained from Cornish Crispa Co, United Kingdom) in the larval stage and weighing 0.2–0.3 g were used to study the virulence of *S. aureus*. Ten randomly picked individuals were used in each experiment for each strain, and all the strains were tested in three independent experiments. *S. aureus* strains were cultured in TSB with appropriate antibiotics at 37°C for 16 h. The cultures were centrifuged at 2500 ×*g* for 5 min and the supernatant was removed. Cells were washed once with PBS, then resuspended in PBS to 1.0 × 10^9^ CFU/ml. The 10 μl of the cell suspension or PBS was injected into the hemocoel of the larvae via the last left pro-leg. Once infected, larvae were incubated at 37°C for 5 days, and the survival rate scored every 24 h. Kaplan–Meier survival analysis was performed on the data and the significance of the difference between the percentage survivals of *G. mellonella* infected with different strains was determined using the Log Rank test. A *p*-value of less than 0.05 was considered statistically significant.

## Results

### Isolation of a *S. aureus* SCV and Characterization of the Auxotrophic Phenotype

A *S. aureus* SCV, SCV445, was selected by growth of *S. aureus* LS-1 on TSB agar plates containing 50 μg/ml kanamycin. This strain exhibited typical SCV phenotypes: pinpoint colonies about 1/10 of the size of the wild-type, which were mannitol fermentation negative and had low coagulase activity and low haemolytic activity (data not shown). SCV445 also had greatly increased resistance to kanamycin compared to the wild-type. The MIC of kanamycin against the wild-type LS-1 strain was 16 μg/ml, while it increased to 256 μg/ml for SCV445. The auxotrophism of SCV445 was determined using the disk diffusion method, and it was found that menadione restored growth but hemin or thymidine did not (data not shown).

### Identification of the Genetic Mutation in SCV445

To identify the genetic basis of the SCV phenotype of SCV445, genes involved in menadione biosynthesis were amplified and sequenced. These included previously identified menadione biosynthetic genes *menA, menB, menC, menD, menE, menF* (Nowicka and Kruk, 2010), a *gerC* locus (also named *hepT-menG-hepS*), and genes with locus tags SAOUHSC_01348 and SAOUHSC_02556. The *gerC* locus has been reported to be involved in menadione biosynthesis in *Bacillus subtilis* (Leatherbarrow et al., 1998), and SAOUHSC_01348 and SAOUHSC_02556 are homologs of a thioesterase-encoding gene that participates in menadione biosynthesis in the cyanobacterium *Synechocystis* sp. PCC6803 (Widhalm et al., 2009). Unexpectedly, no mutations were found in any of these genes when SCV445 was compared to the wild-type strain.

**FIGURE 1:**
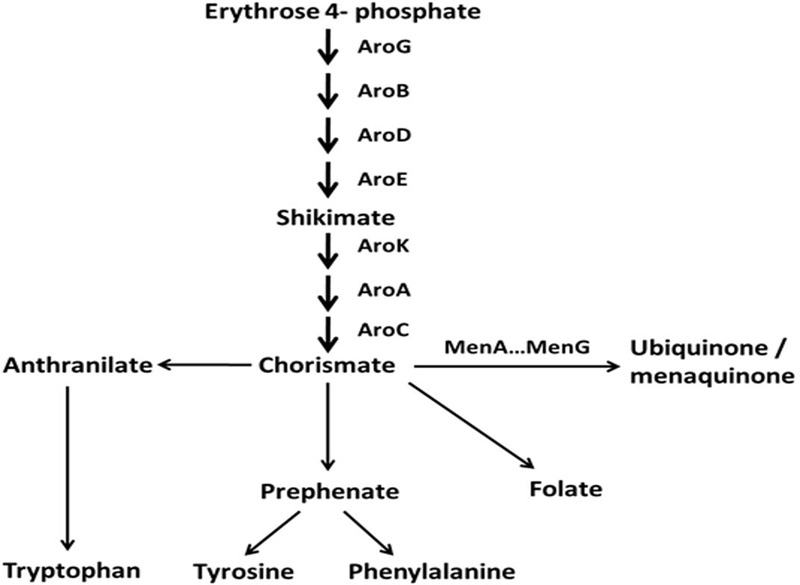
Schematic representation of the shikimate pathway and several downstream biosynthetic pathways in *S. aureus*. The pathway is modified from KEGG (Kyoto Encyclopedia of Genes and Genomes) pathways: phenylalanine, tyrosine, and tryptophan biosynthesis, http://www.genome.jp/kegg/pathway/map/map00400.html.

**FIGURE 2:**
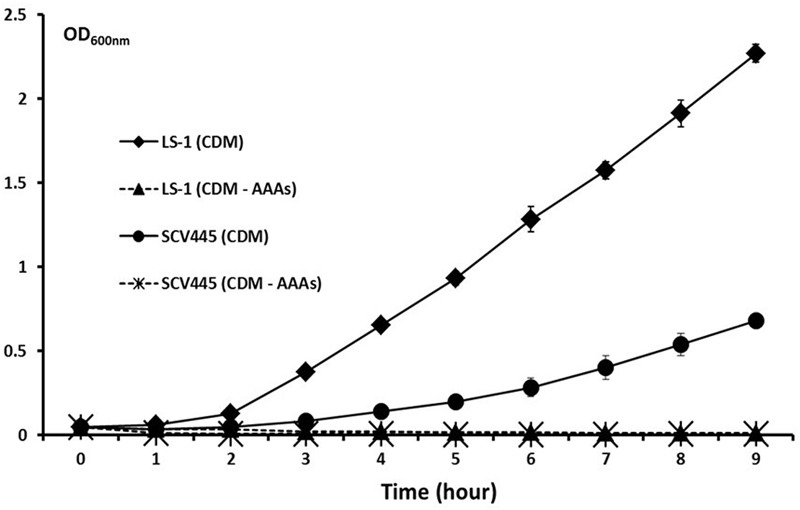
Neither the SCV SCV445 nor the parent strain LS-1 can grow in chemically defined medium lacking aromatic amino acids. Growth of *S. aureus* LS-1 and SCV445 in chemically defined medium (CDM). CDM without aromatic amino acids (CDM – AAAs). Data are presented as the mean of three independent experiments with error bars showing the standard deviations.

**FIGURE 3:**
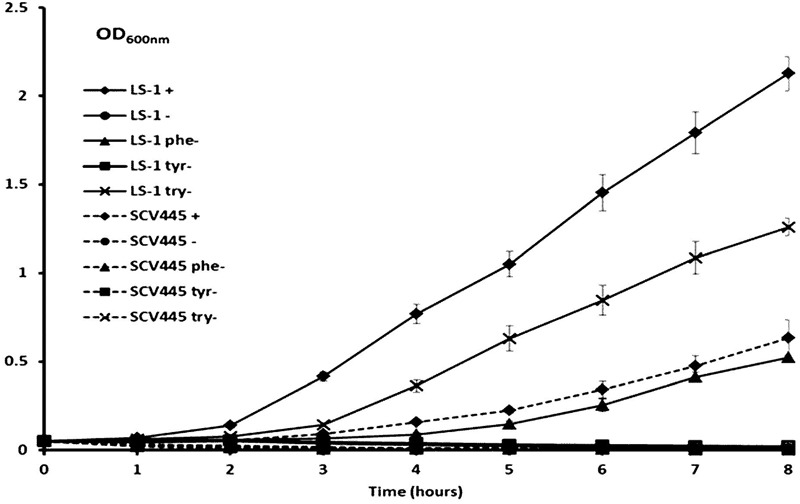
The SCV SCV445 requires all three aromatic amino acids to grow in CDM. Growth of *S. aureus* LS-1 and SCV445 in chemically defined medium (CDM) with or without aromatic amino acid(s). LS-1+ or SCV445+:*S. aureus* LS-1 or SCV445 with the three aromatic amino acids (AAAS). LS-1- or SCV445-:*S. aureus* LS-1 or SCV445 without the three AAAs. LS-1 phe- or SCV445 phe-: bacteria grown in CDM without phenylalanine but with the other two AAAs. LS-1 tyr- or SCV445 tyr-: bacteria grown in CDM without tyrosine but with the other two AAAs. LS-1 try- or SCV445 try-: bacteria grown in CDM without tryptophan but with the other two AAAs. Data are presented as the mean of three independent experiments with error bars showing standard deviations.

To investigate whether a mutation occurred in genes coding for enzymes involved in a pathway upstream of menadione biosynthesis, we investigated the shikimate pathway which is shown in **Figure 1**. The products of the *aro* genes are responsible for the synthesis of chorismate, which is a branching point for several metabolic pathways, including aromatic amino acid biosynthesis, folate biosynthesis and menaquinone biosynthesis. Therefore a mutation in any of the *aro* genes would cause a defect in the other metabolic pathways downstream of chorismate in addition to menadione auxotrophy. Therefore to test the hypothesis that there was a defect in the shikimate pathway, we measured the growth of SCV445 and the wild-type LS-1 in defined medium with or without the aromatic amino acids phenylalanine, tyrosine, and tryptophan. Surprisingly, both SCV445 and LS-1 were unable to grow in the absence of aromatic amino acids (**Figure 2**). To examine this further, we tested the growth of both strains in defined medium in the absence of each of the individual aromatic amino acids. The wild-type strain LS-1 was unable to grow in the absence of tyrosine, while SCV445 failed to grow in the absence of any single aromatic amino acid (**Figure 3**). This finding suggested the presence of a mutation in a gene involved in tyrosine synthesis in the wild-type strain LS-1, while the mutation in SCV445 conferring the SCV phenotype was likely to be in one of the *aro* genes.

**FIGURE 4:**
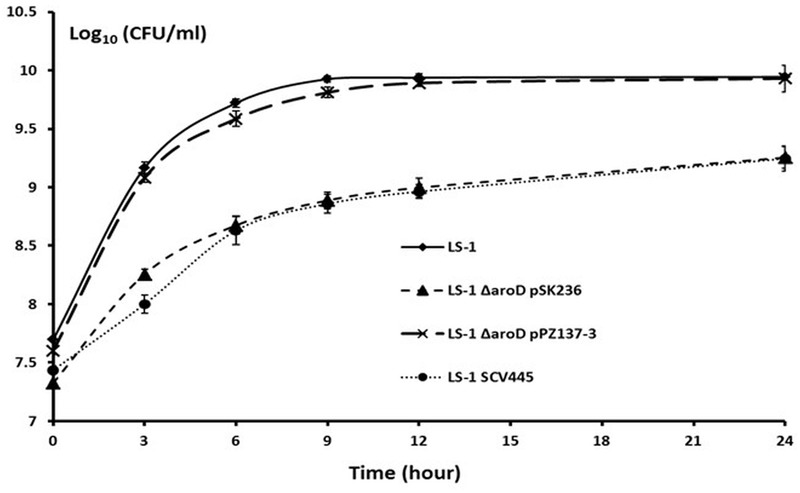
Disruption of *aroD* in *S. aureus* results in a growth defect similar to that of SCV445 and this can be complemented *in trans* by a plasmid borne copy of *aroD*. Viable counts of strains LS-1, LS-1 Δ*aroD* pSK236 (plasmid without an insert), LS-1 Δ*aroD* pPZ137-3 (pSK236::*aroD*) and SCV445 growing in TSB at different time points. Data are presented as the means of three independent experiments with error bars showing the standard deviations.

To locate the mutation in SCV445, the seven *aro* genes were amplified from both LS-1 and SCV445 and sequenced. Sequence alignment between *S. aureus* LS-1 and SCV445 showed that the *aroD* gene in SCV445 had a substitution of 448A > T, resulting in the 149th codon changing from AAA (lysine), to a stop codon TAA. Given that the product of *aroD*, 3-dehydroquinate dehydratase, is essential for the synthesis of chorismate, this mutation was likely to be the cause of both the menadione and the aromatic amino acid auxotrophy in SCV445. To confirm this, we constructed an *aroD* mutant of LS-1 by deleting the sequence beyond nucleotide 447 in the *aroD* gene and adding a TAA stop codon to the truncated gene. The LS-1 *aroD* mutant exhibited the same phenotypes as SCV445, menadione and aromatic amino acid auxotrophy (data not shown) and had a reduced growth compared to LS-1 (**Figure 4**). Expression of *aroD in tran*s from the plasmid pPZ137-3 in the *aroD* deletion strain of LS-1 restored its prototrophic phenotype (data not shown) and growth rate to the same level of LS-1 (**Figure 4**).

### Disruption of *aroD* in *S. aureus* Results in a Greater Capacity to Form Biofilm

**FIGURE 5:**
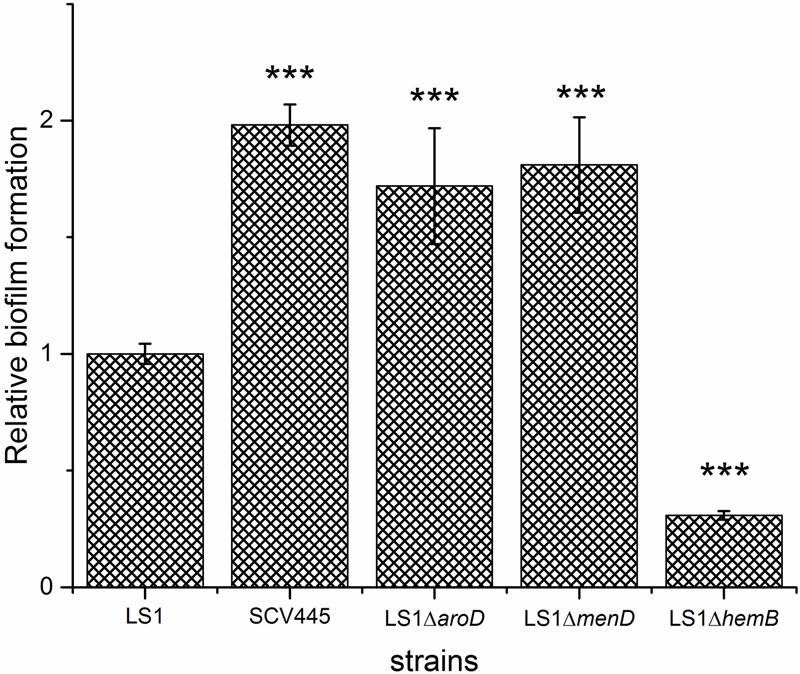
Disruption of *aroD* in *S. aureus* results in a greater capacity to form biofilm. Biofilm formation on plastic plates in BHI containing 1% glucose. Bacteria were added to microtiter wells at an OD_600__nm_ of 0.02 and incubated for 24 h statically at 37°C. The bars represent the mean with error bars showing the standard deviation. ^∗∗∗^*P* < 0.001.

As mentioned, apart from SCVs that arose as a result of triclosan treatment (Latimer et al., 2012) the SCV phenotype in *S. aureus* has generally been reported to be associated with increased biofilm formation (Mitchell et al., 2010a,b). SCV445, an *aroD* and a *menD* deletion mutant of strain LS-1 all had a greater capacity to form biofilms than the LS-1 wild-type strain (**Figure 5**). Given the SCVs are slow growing compared to the wild-type, the increased capacity to form biofilms is remarkable, if the SCVs also grow slowly in the biofilm. Unexpectedly, a *hemB* deletion mutant of LS-1 had a reduced capacity to form biofilms compared to the parent strain (**Figure 5**).

### Rate of Phenotypic Switching from SCV to NCP

In three independent cultures plated out on TSA plates no NCPs were visible after 24 h of incubation, while a total of six large colonies were identified from the three cultures after 48 h of incubation, suggesting phenotypic switching had occurred. Streaking these six colonies on TSA confirmed that they were NCPs that formed normal sized colonies after 24 h incubation. In three independent experiments, the reversion rates were determined to be 2.29 × 10^-8^, 2.83 × 10^-8^, and 1.52 × 10^-7^ per cell per generation giving a mean rate of 6.79 × 10^-8^ per cell per generation.

### Genetic Basis for Phenotypic Switching from SCV to NCP

**FIGURE 6:**
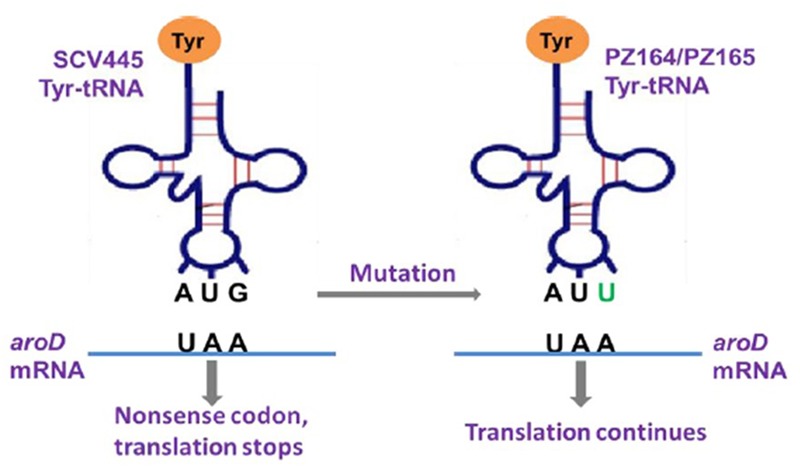
Graphic illustration of the mechanism that a tyr-tRNA mutation restores a functional AroD in PZ164 and PZ165 (Murgola, 1990).

The *aroD* gene was amplified from the six clones that had switched back to NCPs and their sequences analyzed. Four of the NCPs had the same *aroD* sequence as the original wild-type, whereas the other two NCPs (PZ164 and PZ165) retained the same mutation as SCV445. Furthermore there were no other mutations in the gene or the intergenic region of *aroD* in these clones. We reasoned that the only way these clones could have the NCP phenotype was if a functional 3-dehydroquinate dehydratase was still produced despite the presence of the *aroD* mutation. One possible mechanism that could account for this was that these clones may possess a tRNA gene with a mutated anticodon that recognizes the stop codon UAA and therefore allows read through translation of their *aroD* mRNA. There are seven tRNA molecules with a complementary anticodon that, with a single base change, could recognize an UAA stop codon. These are tRNA-tyrosine (UAU), tRNA-tyrosine (UAC), tRNA-leucine (UUA), tRNA-serine (UCA), tRNA-lysine (AAA), tRNA-glutamine (CAA), and tRNA-glutamic acid (GAA). The gene encoding each of these was amplified and sequenced. A mutation was found in the tyr-tRNA gene of both NCPs that had switched from the SCV phenotype and retained the *aroD* mutation. As shown in **Figure 6**, the tyr-tRNA mutation causes an anticodon change from AUG to AUU, which would therefore recognize the stop codon UAA. While both NCPs had the same mutation they were in different copies of the two tyr-tRNA genes in *S. aureus* (corresponding to locus tags SAOUHSC_T00057 and SAOUHSC_T00058 in the genome sequence of strain NCTC8325).

### The Two NCP tyr-tRNA Mutants Have Equivalent Planktonic Growth Characteristics to the Wild-type, Have the Same Capacity to Form Biofilms and Have Restored Aminoglycoside Sensitivity

**FIGURE 7:**
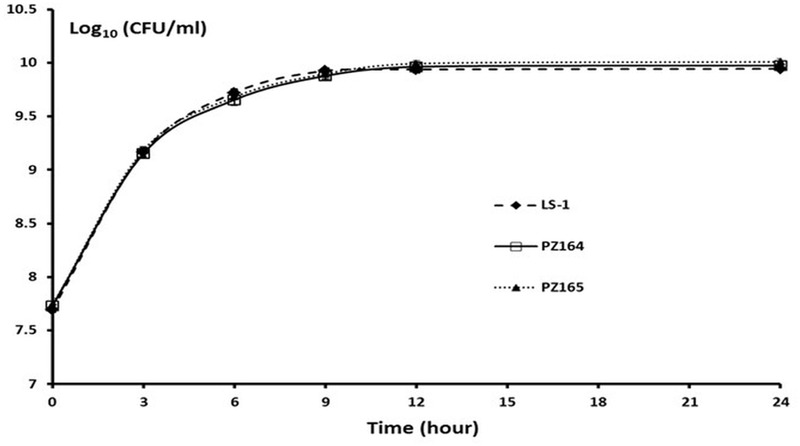
The NCP tyr-tRNA mutants grow at the same rate as the wild-type strain LS-1. Viable counts of strains LS-1, PZ164, and PZ165 growing in TSB at different time points. Data are presented as the means of three independent experiments with error bars showing the standard deviations.

**FIGURE 8:**
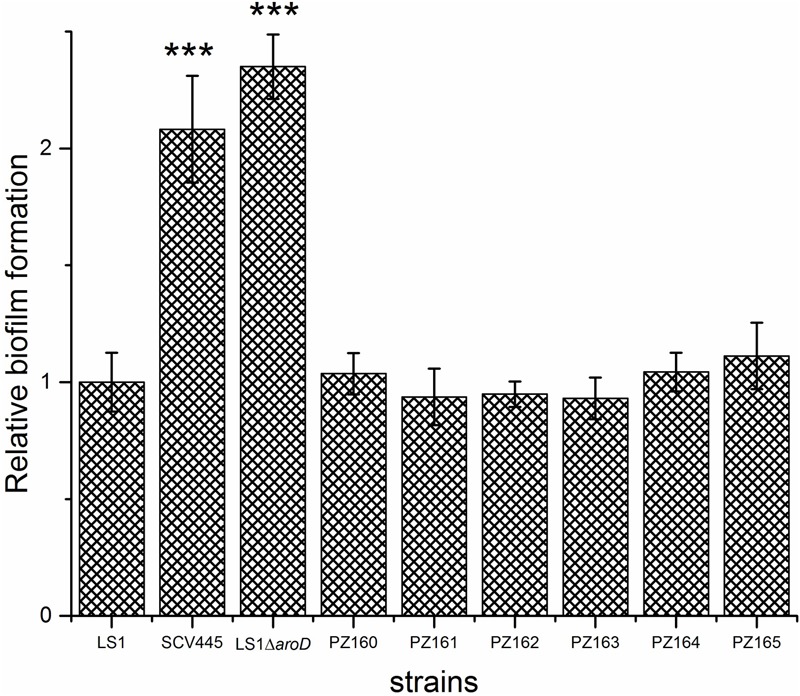
Phenotypic switching from the SCV to the NCP restores the wild-type biofilm phenotype regardless of the genetic mechanism. Biofilm formation on plastic plates in BHI containing 1% glucose. Bacteria were added to microtiter wells at an OD_600__nm_ of 0.02 and incubated for 24 h statically at 37°C. The bars represent the mean with error bars showing the standard deviations. ^∗∗∗^*P* < 0.001.

**FIGURE 9:**
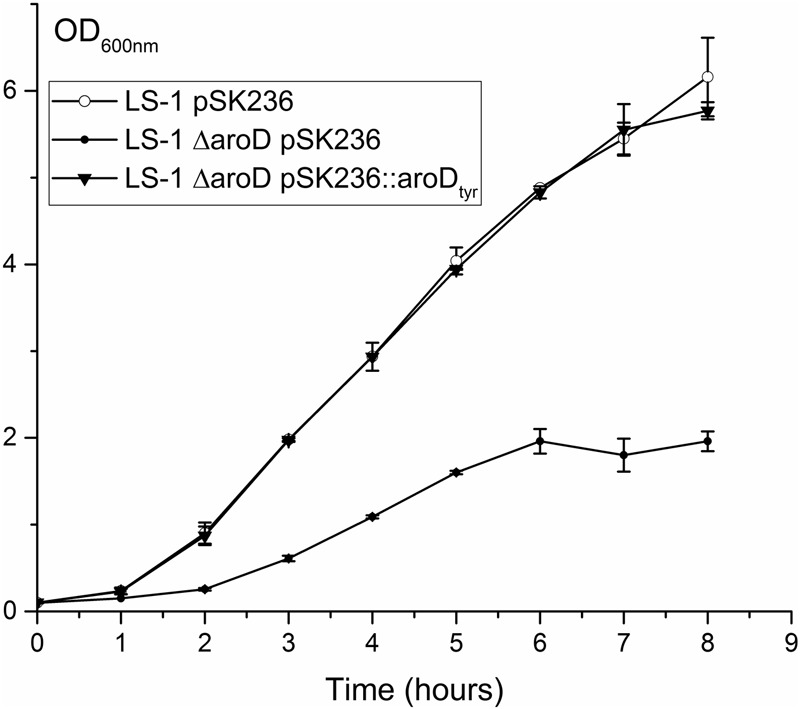
Disruption of *aroD* in *S. aureus* results in a growth defect similar to that of SCV445 and this can be complemented *in trans* by a plasmid borne copy of a synthetic *aroD* gene coding for a K149Y substitution. Growth curves (OD_600__nm_) of strains LS-1, LS-1 *aroD* pSK236 (plasmid without an insert) and LS-1 *aroD* containing pSK236::*aroD_tyr_* growing in TSB at different time points. Data are presented as the means of three independent experiments with error bars showing the standard deviations.

The difference between the 3-dehydroquinate dehydratase produced by the wild-type strain and the two tyr-tRNA mutants was a K149Y substitution. Given the NCP of the two-tRNA mutants, this amino acid substitution must be tolerated, giving an enzyme that retains activity. However, the suppressor tyr-tRNAs in PZ164 and PZ165 should partially repress the translation termination of genes with TAA stop codons, which could affect global gene expression and therefore impact on other phenotypes. To test this possibility we initially examined the susceptibility to kanamycin, planktonic growth characteristics and the capacity of PZ164 and PZ165 to form biofilms. PZ164 and PZ165 had identical sensitivity to kanamycin as the wild-type LS-1 and the four NCP revertants PZ160 – PZ163 (16 μg/ml), similar growth rates to LS-1 (**Figure 7**) and similar capacities to form biofilms as the wild-type and revertants PZ160 – PZ163 (**Figure 8**). To confirm that the 3-dehydroquinate dehydratase with a K149Y substitution, which would be produced in the ochre suppressor strains, accounted for the NCP we complemented the LS-1 *aroD* mutant with a mutated gene encoding this change using plasmid pSK236::*aroD*_tyr._ This *trans* complementation restored the NCP (data not shown) and resulted in similar growth to LS-1 (**Figure 9**).

### The SCV Strains and the Two NCP tyr-tRNA Mutants Are Attenuated in a *Galleria mellonella* Infection Model

**FIGURE 10:**
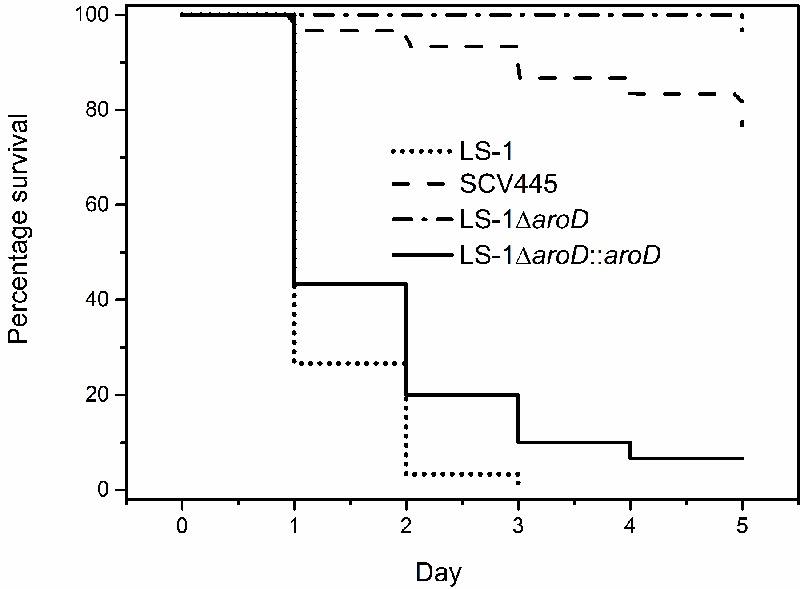
The SCV strains are attenuated for virulence in *Galleria mellonella.* Percentage survival of *G. mellonella* infected with *S. aureus* strains and monitored over 5 days. *G. mellonella* were infected with either the wild-type strain LS-1, or the SCV (SCV445), or an *aroD* mutant of LS-1 (LS-1Δ*aroD*) or an *aroD* mutant of LS-1 complemented with *aroD in trans* (LS-1Δ*aroD::aroD*). Data are presented as the percentages of 30 worms, which were assayed in groups of 10 on three different days.

**FIGURE 11:**
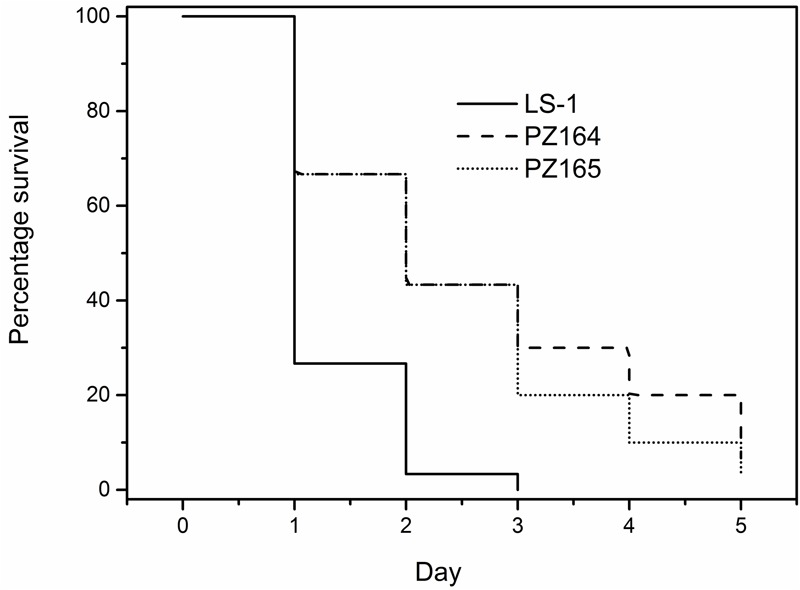
The NCP tyr-tRNA mutants are attenuated for virulence in *Galleria mellonella.* Percentage survival of *G. mellonella* infected with *S. aureus* strains and monitored over 5 days. *G. mellonella* were infected with either the wild-type LS-1, or the NCP tyr-tRNA mutants PZ164 and PZ165. Data are presented as the percentages of 30 worms, which were assayed in groups of 10 on three different days.

The virulence potential of the wild-type LS-1, the SCV, SCV445, the *aroD* mutants and the two tyr-tRNA mutants (PZ164 and PZ165) was evaluated using a *G. mellonella* infection model. **Figure 10** shows a Kaplan–Meier plot for survival of infected *G. mellonella* over time. Compared to the parental strain LS-1, SCV445 and the defined *aroD* mutant showed significantly reduced virulence (*P* < 0.001) and there was no significant difference between SCV445 and LS-1 *aroD* mutant (*P* = 0.171). Complementation with a functional *aroD* gene using plasmid pPZ137-3 restored the virulence of the defined mutant to the same levels as the wild-type. The Kaplan–Meier plot shown in **Figure 11** demonstrates that compared to the parental strain LS-1 both tyr-tRNA mutants have significantly attenuated virulence (*P* < 0.001).

## Discussion

Studies on *S. aureus* SCVs have focused on hemin or menadione auxotrophs, mainly using stable defined *hemB* and *menD* mutants (von Eiff et al., 1997b, 2006; Bates et al., 2003; Sifri et al., 2006). *S. aureus* SCVs are frequently isolated from patients, but the genetic basis of SCV phenotypes is not often characterized (Kahl et al., 2003; Yagci et al., 2013). Nevertheless, those SCVs that have been characterized have demonstrated that mutations in *hemB* or *menD* are not the only mutations that can give rise to the SCV phenotype. For example mutations in *menB* (Lannergård et al., 2008), *menC, menE, menF* (Dean et al., 2014), *hemH* (Schaaff et al., 2003), and *relA*, a gene that encodes guanosine polyphosphate pyrophosphohydrolase/synthetase (ppGpp synthetase) (Gao et al., 2010), give rise to a SCV phenotype. Herein, we report an *S. aureus* SCV with a previously undescribed genotype, an ochre mutation in the *aroD* gene. Although this mutation would result in a defect in the biosynthesis of a number of metabolites, including aromatic amino acids, folate and menaquinone, supplementation of growth medium with menadione alone was sufficient to reverse the SCV phenotype. The finding that a previously undescribed mutation results in menadione auxotrophy, and the SCV phenotype, suggests that there may be an under appreciation of the range of mutations that lead to this phenotype. Indeed, although the novel *aroD* SCV we isolated has similar growth characteristics and capacity to form biofilms as the *menD* SCV, and a reduced virulence potential as reported for the *menD* SCV (Sifri et al., 2006), it is probable that SCVs resulting from different genetic changes will have different pathogenic phenotypes. In support of this hypothesis is our finding that, while the menadione auxotrophic SCVs (due to mutation of either *aroD* or *menD*) had an increased capacity to form biofilms, the *hemB* SCV was less able to form biofilms than the wild-type. Therefore, knowledge of the full range of genetic changes that can lead to the SCV phenotype is necessary if we are to fully understand how these organisms persist during infection and to develop effective treatments against SCVs.

One reason why natural SCVs have not been well studied is that they are thought to be very unstable and quickly undergo phenotypic switching back to the NCP (Becker et al., 2006). However, a relatively low reversion rate of 6.79 × 10^-8^ per cell per generation was determined for the *aroD* SCV, SCV445, described in this study. In addition, a similar reversion rate of 1.8 × 10^-8^ per cell per generation was reported for a clinically isolated *S. aureus menB* mutant (Lannergård et al., 2008) and more recently for *in vitro* generated gentamicin resistant SCVs (Vestergaard et al., 2016). These mutation frequencies are within the range of those reported for spontaneous rifampicin resistant *S. aureus* mutants (between 3.5 × 10^-7^ and 1.10 × 10^-8^ per cell per generation) thus these data suggest that spontaneously formed SCVs are as stable as the NCP, and that phenotypic switching occurs as frequently as other spontaneous mutations. This is also consistent with the findings reported by Edwards (2012) that the formation of SCVs from NCP cells is a natural process during replication rather than a consequence of selective pressure, and that SCV-NCP switching occurs spontaneously during bacterial replication in the absence of selective pressure (Edwards, 2012). Given that by our reasoning SCVs are not particularly unstable, why is it frequently reported that they are? This is probably due to the fact that upon phenotypic switching of a cell from the SCV to the NCP, the NCP cell replicates much more rapidly than the SCV cells, quickly giving rise to a very high proportion of NCP cells within a culture. This may explain the higher SCV reversion rates of 1.76 × 10^-6^ and 1.21 × 10^-6^ per cell per generation observed by Dean et al. (2014), where SCVs were serially passaged in broth without selection for 3–10 days.

It has been assumed that phenotypic switching between the SCV phenotype and the NCP was due to reversion of the mutation that initially led to the SCV phenotype. Indeed until fairly recently studies on the genetic basis for phenotypic switching of *S. aureus* SCVs to NCP have been consistent with this assumption (Lannergård et al., 2008; Dean et al., 2014). More recently Vestergaard et al. (2016) have found new pathways that compensate for the growth fitness of gentamicin resistant SCVs, however, they did not investigate what effect these mutations had on other phenotypes such as biofilm formation and virulence (Vestergaard et al., 2016). We have found that phenotypic reversion can occur via the generation of tRNA-Tyr ochre suppressors, which has not been described previously for SCVs. These findings demonstrate that there are a number of possible genetic events that can lead to phenotypic switching to the NCP apart from the widely reported reversion of the original mutation. Such NCPs may have different phenotypes compared to the wild-type strain and the SCV from which they were derived. In support of this hypothesis is our finding that while the two NCP tyr-tRNA mutants arising from SCV445 had many of the phenotypes of the wild-type strain LS-1, they had reduced virulence in the *G. mellonella* infection model. Given that the reduced virulence of SCVs is associated with their ability to persist in the host, it is tempting to speculate that the tyr-tRNA NCPs might have more potential to persist in the host than the wild-type strain and therefore cause distinct clinical problems from either the wild-type NCP and the SCV.

## Author Contributions

PZ, JW, and AO performed the experiments. PZ, JW, AO, and SN planned the experiments and participated in the interpretation of data and in the writing of the manuscript.

## Conflict of Interest Statement

The authors declare that the research was conducted in the absence of any commercial or financial relationships that could be construed as a potential conflict of interest.
