# Supplementary material for: An aroD Ochre Mutation Results in a Staphylococcus aureus Small Colony Variant That Can Undergo Phenotypic Switching via Two Alternative Mechanisms
Source: Front Microbiol. 2017 May 31;8:1001. doi: 10.3389/fmicb.2017.01001 (PMC5449664; doi:10.3389/fmicb.2017.01001)
Supplement: Supplementary file 1 [file Table_1.DOCX]

Table S1: Oligonucleotides used in this study; Underline: restriction endonuclease sites; dotted underline: *att*B tails used for lambda recombination with *att*P sites.

**Name Oligonucleotide sequence 5’-3’ Restriction sites**

P121 GACATTTGCAGCATATAGAGG

P125 ACTGAAGGATGATTCGTATATTAG

P126 GCATCAGTTTTCTTTAACCA

P128 CACCCATTGAATATCCAAAC

P130 GACGCAAGTGACTTAATATCAT

P131 TGATGAAGTATTCCATTTAAGTGC

P132 CCTTTATTTAGGTATCCGTCGT

P133 AATGCTGGGCGTACTCTTTTAC

P138 TCGTAAGTACAATAAAGGC

P139 ATTTGCTCGTCCACATC

P140 ATCATCTTCGAGTTGTTGG

P141 TGGTGCGCCTACACTTC

P142 GGCAGTGAAAGACCTGTC

P143 TTTCAGATAACAATGTGGC

P144 TTATTGAACAACGATGG

P145 CGGTTTTGGAATAATACG

P146 TTGCTGAAATGATTGACGC

P147 TCGCTGGAAGGGATATTAC

P156 CCTCTAATTGATCCTGGTGAAG

P157 TTGCTGCAATGCACCTTG

P159 TGAATAATGTAAATGAAGTAAAGG

P160 GTATCCAACAGATTTAACGGGTAC

P166 GACGCTATGGTTCGTTTTGACTC

P167 CTGATGTGTCCTGAACCTCGG

P168 GAACCGTTTGGCTTACCTGC

P169 CAATCACTTCAAGCCCTTGATTC

P170 AAGCGTTAGGTTGAATGTATGAG

P171 TAACAATCGTTAAGTGCAGTATACC

P172 GTCGAAAGCACCGTTGAAGC

P173 CATTCCGTGATTTGGAGCATC

P174 GAGTAGCTTCGCTTAATGTTTCAG

P175 GTATCAGGTGGTAAACGTCCG

P176 CACTTGACATTGAAATACCCCC

P177 AAGTCGGTACAAATGTGCAGAC

P178 TTATTTGCTAAAGTTGCGATACG

P179 GCAATAACAATATCCAGCTTCG

P180 CAGTCGTGTTGTTGAAATAGGTG

P181 GTGTTGGCGGTAAAGTGGG

P182 CCAAAATTGTAAAGTGTCTCTCCTAG

P183 GCAATCATGTTGGCGTCG

P189 CAACATGAGCAGTAATACAGCG

P190 CAGAAGCAGGTACAAAACACG

P192 GGCGTCTAGATTAGTATAAAGTCACTTGTGC XbaI

P193 CCAAGGATCCGAAATGAGGGATTCACTATG BamHI

P194 *GGGGACAAGTTTGTACAAAAAAGCAGGCT*GTCGAAAGCACCGTTGAAGCAAG

P195 CCGCCTCGAGTTAAAAAAATATAAATTGC XhoI

P196 CCGCCTCGAGTAAAATGAATTAGGTTGCTT XhoI

P197 *GGGGACCACTTTGTACAAGAAAGCTGGGT*GCTAAGTCACCTGAACAAGTTG

P207 GGTAGGAGGCTTTTGTTATGC

P208 GATGTTATGGAGGACTGGTGG

P209 AAGAAGGTAGAGGCGTAGCG

P210 GTCACAATTATCTGGGGTAGCC

P211 CATGCCGAACACAGAAGTTAAG

P212 CTGAGCCAGGATCAAACTCTC

P213 CGAGACCGCCATTATTATTACC

P214 ACGATTAGGTCATGCAGATGTAC

P215 CATGCCGAACACAGAAGTTAAG

P216 TTACACCTATACCTCATTCCAGG

P217 GATCCCGCTAGTCTCCACC

P218 ATGTGGAGCTGACGAATACTAATC

P219 GCACGTATAATGATGATTTTCAGC
