# Supplementary material for: An aroD Ochre Mutation Results in a Staphylococcus aureus Small Colony Variant That Can Undergo Phenotypic Switching via Two Alternative Mechanisms
Source: Front Microbiol. 2017 May 31;8:1001. doi: 10.3389/fmicb.2017.01001 (PMC5449664; doi:10.3389/fmicb.2017.01001)
Supplement: Supplementary file 2 [file Table_2.DOCX]

**Table S2: Constituents of the chemically defined medium used for growth studies.**

Contains weight to volume 0.5% glucose, 0.7% K_2_HPO_4_, 0.2% KH_2_PO_4_, 0.04% Na_3_ citrate.2H_2_O, 0.005% MgSO_4_, 0.1% (NH4)_2_SO_4_, 0.0001% thiamine, 0.00012% niacin, 0.0000005% biotin, 0.000025% Ca pantothenate, 0.05% L-arginine, 0.01420325% L-asparagine·H_2_O, 0.009% L-aspartic acid, 0.0125% L-cystine, 0.01% L-glutamic acid, 0.005% glycine, 0.00375% L-histidine, 0.005% hydroxy-L-proline, 0.0125% L-isoleucine, 0.0125% L-leucine, 0.01% L-lysine·HCl, 0.00375% L-methionine, 0.00375% L-phenylalanine, 0.008% L-proline, 0.0075% L-serine, 0.005% L-threonine, 0.00125% L-tryptophan, 0.0057993% L-tyrosine, 0.008% L-valine, 0.0005% adenine, 0.0005% guanine, 0.0005% cytosine, 0.002% thymine and 0.02% uracil.
