# Supplementary material for: An aroD Ochre Mutation Results in a Staphylococcus aureus Small Colony Variant That Can Undergo Phenotypic Switching via Two Alternative Mechanisms
Source: Front Microbiol. 2017 May 31;8:1001. doi: 10.3389/fmicb.2017.01001 (PMC5449664; doi:10.3389/fmicb.2017.01001)
Supplement: Supplementary file 3 [file Table_3.DOCX]

###### Table S3: Oligonucleotides used for the amplification and sequencing of the menadione biosynthetic genes, chorismate biosynthetic genes, *tyrA* and tRNAs in LS-1, SCV445 or the NCP tyr-tRNA mutants.

| Category | Gene | Locus tag in  *S. aureus* NCTC8325 | Primers used for amplification | Primers used for sequencing |
| --- | --- | --- | --- | --- |
| Genes coding for proteins involved in menadione biosynthesis | *menA* | SAOUHSC_00980 | P121, P141 | P121, P125, P138, P139, P140 |
|  | *menF* | SAOUHSC_00982 |  |  |
|  | *menD* | SAOUHSC_00983 |  |  |
|  | *menD* | SAOUHSC_00983 | P142, P130 | P142, P143, P144, P145, P146 |
|  | *menH* | SAOUHSC_00984 |  |  |
|  | *menB* | SAOUHSC_00985 |  |  |
|  | *menC* | SAOUHSC_01915 | P131, P132 | P131, P132, P147, P160 |
|  | *menE* | SAOUHSC_01916 |  |  |
|  | *gerC* | SAOUHSC_01486 | P156, P157 | P156, P157 |
|  | *menG* | SAOUHSC_01487 |  |  |
|  | *menG* | SAOUHSC_01487 | P133, P159 | P133, P159 |
|  | *hepS* | SAOUHSC_01488 |  |  |
|  |  | SAOUHSC_01348 | P166, P167 | P167 |
|  |  | SAOUHSC_02556 | P168, P169 | P168, P169 |
|  | | | | |
| Genes coding for proteins involved in tyrosine biosynthesis | *tyrA* | SAOUHSC_01364 | P189, P190 | P189, P190 |
|  | | | | |
| Category | Gene | Locus tag in  *S. aureus* 8325-4 | Primers used for amplification | Primers used for sequencing |
| Genes coding for proteins involved in  chorismate biosynthesis | *aroA* | SAOUHSC_01481 | P178, P179 | P178, P179, P180 |
|  | *aroB* | SAOUHSC_01482 |  |  |
|  | *aroB* | SAOUHSC_01482 | P181, P182 | P181, P182, P183 |
|  | *aroC* | SAOUHSC_01483 |  |  |
|  | *aroD* | SAOUHSC_00832 | P172, P173 | P172, P173 |
|  | *aroE* | SAOUHSC_01699 | P174, P175 | P174, P175 |
|  | *aroG* | SAOUHSC_01852 | P170, P171 | P170, P171 |
|  | *aroK* | SAOUHSC_01635 | P176, P177 | P176, P177 |
|  | | | | |
| tRNA genes | GlutRNA | SAOUHSC_T00018 | P207, P208 | P207 |
|  | SertRNA | SAOUHSC_T00048 | P209, P210 | P209 |
|  | GlutRNA | SAOUHSC_T00019 | P213, P214 | P213 |
|  | Lys tRNA | SAOUHSC_T00035 | P211, P212 | P211 |
|  | LeutRNA | SAOUHSC_T00030 |  |  |
|  | GlntRNA | SAOUHSC_T00016 | P215, P216 | P215, P216, P217 |
|  | Tyr tRNA | SAOUHSC_T00057 |  |  |
|  | SertRNA | SAOUHSC_T00051 |  |  |
|  | SertRNA | SAOUHSC_T00052 |  |  |
|  | LeutRNA | SAOUHSC_T00032 |  |  |
|  | Lys tRNA | SAOUHSC_T00036 |  |  |
|  | Lys tRNA | SAOUHSC_T00037 | P218, P219 | P218 |
|  | GlntRNA | SAOUHSC_T00017 |  |  |
|  | Tyr tRNA | SAOUHSC_T00058 |  |  |
|  | GlutRNA | SAOUHSC_T00020 |  |  |
